# Hematological abnormalities and associated factors among patients with thyroid hormone dysfunction at the University of Gondar Comprehensive Specialized Hospital, Northwest Ethiopia

**DOI:** 10.1371/journal.pone.0322748

**Published:** 2025-05-02

**Authors:** Befikad Mandefro, Amanuel Kelem, Zenebe Abebe Gebreegziabher, Yemataw Gelaw, Elias Shiferaw, Tiruneh Adane

**Affiliations:** 1 Department of Medical Laboratory Sciences, Asrat Woldeyes Health Science Campus, Debre Berhan University, Debre Berhan, Ethiopia; 2 Department of Epidemiology and Biostatistics, School of Public Health, Asrat Woldeyes Health Science Campus, Debre Berhan University, Debre Berhan, Ethiopia; 3 Department of Hematology and Immunohematology, School of Biomedical and Laboratory Sciences, College of Medicine and Health Sciences, University of Gondar, Gondar, Ethiopia; Sai Gosavi Specialty Clinic / Nano Hospitals Bangalore / Saraswati Specialty Clinic, INDIA

## Abstract

**Background:**

Thyroid hormones substantially influence the metabolism and production of blood cells; as a result, blood disorders are frequently seen among patients with thyroid hormone disorders. Therefore, this study aimed to assess hematological abnormalities and associated factors among patients with thyroid hormone dysfunction.

**Methods:**

A hospital-based cross-sectional study was conducted from March 12/03/2022 to May 26/05/2022 among consecutive selected 308 study participants at the University Gondar Comprehensive Specialized Hospital, Northwest Ethiopia. A structured questionnaire and data extraction sheet were used to collect socio-demographic and Clinical data, respectively. For complete blood cell count analysis venous blood was collected and analyzed by Beckman-coulter DXH-800 hematology analyzer. Data was entered by Epi data version 3.1 and analyzed by Stata version 14. Binary and multivariable logistic regressions were done to identify associated factors of hematological abnormality. A P-value less than 0.05 was considered statistically significant.

**Results:**

The overall magnitude of anemia, leukopenia, thrombocytopenia, thrombocytosis, leukocytosis, and polycythemia was 26.3%, 5.5%, 2.6%, 2.3%, 2.3%, and 1.3%, respectively. Hypothyroidism (AOR = 2, 95% CI:1.0–3.6), alcohol consumption (AOR = 4, 95% CI: 1.7–9.2), meat consumption (AOR = 4, 95% CI: 1.6–10.4), vegetable consumption (AOR = 2.5, 95% CI:1.1–5.5) and febrile illness (AOR = 2.6, 95% CI:1.3–5.4) were found to be associated with anemia.

**Conclusion:**

Anemia was a moderate public health problem among thyroid dysfunction patients, mainly normocytic normochromic anemia was the most common type of anemia, leukopenia was second major hematological abnormality. Hypothyroidism, alcohol consumption, meat consumption, vegetable consumption, and febrile illness were associated with anemia. Thus, all patients with thyroid dysfunction should have regular anemia screenings, particularly those with important risk factors. This could aid in the early identification and efficient treatment of anemia, improving the patients’ quality of life.

## Introduction

The thyroid gland is the most important hormone-producing organ located in front of the neck that wraps around the windpipe (trachea) [1]. The gland is responsible for producing thyroxine (T4) and triiodothyronine (T3) [2]. Thyroid hormones (TH) are essential for growth and development and play a critical role in energy homeostasis [3]. It influence increased oxygen consumption, which plays a crucial role in optimal growth, brain development, and sexual maturity [4].

Thyroid dysfunction which can be hypo or hyperthyroidism is a common endocrine disorder affecting around million people worldwide, and it is assumed that more than half are unaware of their disorder [2]. The prevalence of thyroid dysfunction varies widely among different populations, with estimates ranging from 4% to 10%, and it can be as high as 18% in older adults [5]. It has also been indicated that the disorder is more common in women than in men with an annual rate of 4.1 and 0.6 per 1000, respectively [2]. In Ethiopia, the situation is particularly striking, with a reported prevalence of thyrotoxicosis at 43.7%. This indicates a significant public health concern, highlighting the need for awareness and appropriate management strategies in various demographic groups [6]. Additionally it has been indicated that Thyroid swellings are frequent in Ethiopia, especially in the highland areas, with a prevalence of 18–30%, [7].

thyroid gland modulate the metabolism, differentiation, and proliferation of almost every cell in the body [8] including red blood cell (RBC), platelet and white blood cell (WBC) synthesis [9,10]. Previous finding indicated that thyroid dysfunction affects normal blood cell production resulting in hematological abnormality. The pathophysiology of thyroid dysfunction in hematological abnormalities has not been well stated, though some literature try to elaborate [11–13]. Even if the mechanism is not well mentioned, hematological abnormalities such as anemia, leukocytopenia, leukocytosis, thrombocytopenia, and thrombocytosis are common findings in thyroid dysfunction patients [10], which can also be justified by the presence normal hematological values in individuals with functional thyroid hormone (euthyroidism) [14].

In terms of WBC and platelet, hypothyroid patients have a slightly reduced total WBC count, neutropenia, and thrombocytopenia [15]. Furthermore, hyperthyroid patients have raised, normal, or slightly lowered total WBC count, with a relative decrease in neutrophils and an increase in eosinophils and mononuclear cells. Nonetheless, studies found hyperplasia in all myeloid cell lines in hyperthyroidism and hypoplasia in hypothyroidism [16]. With regard to lymphocytes,T_3_ has been shown to be a prerequisite for normal B-cell production in the bone marrow through its regulation of pro-B-cell proliferation [17].

Worldwide anemia was the most prevalent hematological abnormality among thyroid dysfunction patients, particularly in Ethiopia prevalence rate of 66.5% was indicated in thyroid dysfunction patients [12,18,19]. Particularly in Ethiopia the prevalence of anemia among hypothyroidism was 57.5 but in hyperthyroidism it was 53.3 [1]. The type of anemia was different between the findings, that could be normochromic-normocytic, microcytic-hypochromic or macrocytic normochromic anemia [20]. Moreover, the associations between thyroid hormone levels with WBC and platelet, is inconsistent [21,22]. In spite of showing the association between thyroid disorder and hematological abnormality, there was variability between reported studies. In addition, only a limited studies have been done in Ethiopia on the alteration of hematological abnormalities in thyroid dysfunction patients. Furthermore, previous studies did not thoroughly assess the associated factors. In this context, studying the extent of hematological abnormalities and associated factors among thyroid dysfunction patients will add a significant value to patients, the community and the scientific society. So that, this study aimed to assess hematological abnormalities and associated factor in thyroid hormone dysfunction patients at University of Gondar Comprehensive Specialized Hospital North west Ethiopia.

## Materials and methods

### Study area, study design, and period

A hospital-based cross-sectional study was conducted at the University of Gondar Comprehensive Specialized Referral Hospital from March 12/03/2022 to May 26/05/2022. The hospital is located in the central Gondar zone, Gondar town, Amhara Regional State. It is located 738 km far from the capital city of Ethiopia, Addis Ababa, and 180 km far from the capital city of the Amhara region, Bahir Dar. The town has 2133 meters elevation above sea level. According to the national reports conducted by the Central Statistical Agency of Ethiopia, Gondar City has a total population of 360,600. The town has one referral hospital, five government health centers, and more than 45 private clinics. The hospital provides different medical services for more than 7 million people in the zones and people of the neighboring zones through other wards and outpatient departments [23].

### Source of population, study population and variables

The source population was all patients with thyroid hormone dysfunction who attended the University of Gondar Comprehensive Specialized Referral Hospital and patients with thyroid hormone dysfunction who attended during the data collection period, fulfilled the inclusion criteria, and were considered a study population. At the same time, the magnitude of hematological abnormalities (anemia, polycythemia, leukopenia, leukocytosis, thrombocytopenia, and thrombocytosis) were the dependent variable. Socio-demographic characteristics of the respondents (age, gender, marital status, residence, educational status, occupation), behavioral characteristics of patients (smoking status, alcoholism, meat consumption, vegetable consumption, iodine salt consumption), and Clinical characteristics of respondents (Parasitic infection and types of thyroid dysfunction, febrile illness) were taken as an independent variable. A total of 308 consecutively selected thyroid dysfunction patients were included in this study. Patients take non-steroidal anti-inflammatory drugs, phenazopyridine, and penicillin (before 3monthes). Pregnant women, recently transfused patients, those patients having surgery (In the last three months), and active traumatic bleeding were excluded from the study.

### Operational definitions

Hematological Abnormalities: This Hematological Abnormalities include Anemia, Polycythemia, Leukopenia, Leukocytosis, Thrombocytopenia and Thrombocytosis

#### Anemia.

The Hb concentration within RBC is lower than normal, Hb levels <12.0 g/dL in women and < 13.0 g/dL in men. Morphological classification of anemia based on MCV: microcytic (MCV < 80 fl), normocytic (MCV between 80 and 100 fl), and macrocytic (MCV > 100 fl) [24].

#### Polycythemia.

Is defined as an elevated Hct count, more than 55.3% and 50.1% for males and females, respectively [25].

#### Thyroid dysfunction.

A condition of hormonal disorder commonly, hypothyroidism (T4 < 6.09 μg/dL and T3 < 0.87 ng/dL whereas TSH > 5.6μIU/L) and Hyperthyroidism (TSH < 0.34μIU/L but T3 > 1.78 ng/dL and T4 > 12.23μg/dL) [26].

#### Leukopenia.

A low level of WBC in the blood, defined as a WBC count below 3 x 10^3^ cells/µl [25].

#### Leukocytosis.

Leukocytosis is the broad term for an elevated WBC count, typically above 11.2 x10^3^ cell/µl, on a peripheral blood smear collection [25].

#### Thrombocytosis.

When the platelet count is higher than 399 x10^3^ cells/ µl [25].

#### Thrombocytopenia.

When blood platelet count below 90x10^3^ cells/ µl [25].

#### Smoker.

Anyone who has smoked at least 100 cigarettes in their lifetime and now smokes every day/ regular smoker [27,28].

#### Alcohol user.

Alcoholc consumption was defined as a minimum of 1–2 drinks per day for women and 1–4 drinks per day for men [29,30].

### Data collection and laboratory methods

Socio-demographic characteristics, behavioral characteristics of patients were collected using a structured questionnaire via a face-to-face interview technique, and Clinical characteristics of respondents were collected using data extraction sheet. Before the interviews, the questionnaires were pre-tested by taking 10% [31] of the total sample size at Tibebe Gihon Hospital Bahir Dar, Ethiopia. Three (3 ml of venous blood were collected by trained laboratory professionals aseptically from thyroid dysfunction patients using EDTA tubes for CBC analysis. The collected whole blood was analyzed using the Beckman-coulter 800 BC Hematology Analyzer (United States of America). Pea-size (1 gm) fresh stool samples from each respondent were collected with dry, clean, and leak-proof containers. Wet mount stool preparation was used to prepare the stool for examination. Finally, parasites were examined under 10x and 40x microscope objectives. Both thin and thick, Blood films, were made and left to air dry. Methanol was used to fix the thin blood film, and 10% Giemsa stain was applied to both the thin and thick blood films for ten minutes. The slides were cleaned with clean water, allowed to air dry, and then viewed under a microscope by lab technologists. Malaria was ruled out when thick blood films were found to be negative following the examination of 100 fields using a 100x oil immersion objective. Prior to running the patient’s sample, quality control reagents were run to assess the automated hematology analyzer performance. All quality control reagents manufacturer’s instructions were strictly followed, and samples and reagent integrity were routinely examined. Stool samples were examined for quality upon receipt, and the standard saline solution quality was examined. Known malaria positive and negative slides were used to assess the Giemsa stain quality. Using the University of Gondar comprehensive specialized hospital laboratory standard operating procedures (SOPs), the pre-, analytical, and post-analytical phases of quality assurance were rigorously followed.

### Data analysis

The data were entered into Epi data version 3.1 and analyzed by Stata version 14. Chi-square and Fisher’s exact tests assessed the associations between categorical variables. Moreover, Binary and multivariable logistic regression was used to measure the association between the hematological abnormality and associated factors. Both crude odds ratio (COR) and adjusted odds ratio (AOR) with the corresponding 95% confidence interval (CI) were calculated to measure the strength of association between variables. The Shapiro-Wilk test was used to test the normality of continuous variables, whereas the Hosmer-Lemeshow test checked the model fitness. Finally, a P-value <0.05 was considered statistically significant.

### Ethical approval and consent to participate

Ethical approval for the study was granted by the Ethics Review Committee of the School of Biomedical and Laboratory Science, College of Medicine and Health Science, University of Gondar, under reference number SBMLS/190/2022. Subsequently, a permission letter was obtained from the medical director of the University of Gondar Comprehensive Specialized Hospital. Prior to commencing data collection, data collectors were instructed to seek permission from participants, explain the study’s purpose, significance, and benefits, and address any questions to ensure informed consent. Written consent was then acquired from all participants, with the option to decline participation. For participants under the age of 18, consent was obtained from their parents or guardians. Where the research ethics committee or Institutional Review Board (IRB) waived the requirement for parental consent, this was explicitly noted and justified according to ethical standards. To protect participant confidentiality, data were anonymized using codes, and access was restricted to authorized personnel only. Any abnormal laboratory results were communicated to healthcare professionals to ensure appropriate management and treatment.

## Results

### Socio-demographic characteristics of study participants

A total of 308 participants were involved in this study. Of them, 275 (89.29%) were females. Most study participants (156; 50.7%) were above 45 years old, followed by 31–45 (116; 37.6%). The mean age was 46 ± 11.96 SD years. Most of the respondents (198; 64.29%) were rural residents, and more than half were unable to read and write (166; 53.90%), showed in Table 1.

**Table 1:** Socio-demographic characteristics of thyroid hormone dysfunction patients at the University of Gondar Comprehensive Specialized Hospital, Ethiopia, from March to May 2022 (n = 308).

Variable	Category	Frequency(n)	Percentage (%)
Age/years	17–30	36	11.67
	31–45	116	37.69
	>45	156	50.65
Sex	Male	33	10.71
	Female	275	89.29
Marital statues	Married	158	51.30
	Unmarried	59	19.16
	Divorced	64	20.78
	Widowed	27	8.77
Educational statues	Unable to read and write	165	53.57
	Primary school	74	24.03
	Secondary school	60	19.48
	Certificate and above	9	2.92
Residence	Rural	198	64.29
	Urban	110	35.71
Occupational statues	Farmer	174	56.49
	Civil servants	33	10.71
	Housewife	46	14.94
	private worker	55	17.86

### Behavioral and clinical characteristics of the study participants

Eight (2.6%) of respondents were smokers, 41 (13.3) were iodine salt consumers. Two-thirds (61.36%) of respondents had hyperthyroidism. The result of the stool examination showed that 11 (3.6%) participants had intestinal parasite infections. The most common parasite was Ascaris *lumbricoides* (6;1.9%), followed by Hook warm (3; 1%). In blood film examination, 4 (1.30%) of the patients had malaria; all of them were diagnosed with P. *falciparum* (Table 2).

**Table 2:** Behavioral and clinical characteristics of thyroid hormone dysfunction patients at the University of Gondar Comprehensive Specialized Hospital, Ethiopia, from March to May 2022 (n = 308).

Behavioral and feeding characteristics of respondents			
Variable	Category	Frequency	Percentage (%)
Smoking status	Yes	8	2.6
	No	300	97.4
Iodin salt consumption	Yes	41	13.3
	No	267	86.7
Alcohol consumption	Yes	44	14.3
	No	264	85.7
Vegetable consumption	Yes	126	41.
	No	181	59
Meat consumption	Yes	103	33.7
	No	203	66.3
**Clinical characteristics of thyroid dysfunction patients**			
Febrile Illness	Yes	57	18.5
	No	251	81.5
Thyroid dysfunction	Hypothyroidism	119	38.6
	Hyperthyroidism	189	61.4
Intestinal parasite	Yes	11	3.6
	No	297	96.4
Types of Intestinal parasite	*A. lumbricoides*	6	1.9
	Hook warm	3	1
	*G. lamblia*	2	0.7
Malaria parasite	Yes	4	1.3
	No	304	98.7

### Hematological profiles of thyroid dysfunction patients

Normality of the data was checked using Pearson’s coefficient of skewness and kurtosis. For all hematologic abnormalities, the skewness values were above 0, indicating a right-skewed distribution. As a result, the median with interquartile range was used as a measure of central tendency. The median (IQR) Hb value of the overall study participants was 14 g/dL (2.2), WBC 5.3 × 10^3^/µL (2.5), RBC 4.63 × 10^6^/µL (0.6) and platelet 228000 (95). The median (IQR) Hb value of hypothyroidism was 13.6 g/dL (2.3) and hyperthyroidism 14.4 g/dL (1.5), The median (IQR) values of MCHC, MCH, Hct, WBC, RBC, and platelet among hypothyroidism and hyperthyroidism patients were also different. The result indicated that MCHC 34.9% (1.5) versus (Vs)s 35.1% (1.1), MCH 30.3 pg (2.7) Vs 38.8 pg (2.20), Hct 39.3% (5.4) Vs 40.4% (4.4), WBC 5.4 × 10^3^/µL (2.7) vs 5.1 × 10^3^/µL (2.5), RBC 4.6 × 10^6^/µL (0.7) vs 4.65 × 10^6^/µL (0.6) and platelet 228 (105) vs 228 (92) respectively (Table 3).

**Table 3:** Hematological profile of thyroid hormone dysfunction patients at the University of Gondar Comprehensive Specialized Hospital, Ethiopia, from March to May 2022 (n = 308).

Parameters	Total (n = 308)	Hypothyroidisms (n = 119)	Hyperthyroidisms (n = 189)
	Median (IQR)	Median (IQR)	Median (IQR)
Hb: g/dl	14 (2.2)	13.6 (2.3)	14.4 (1.5)
RBC:10^6^/µl	4.63 (0.6)	4.6 (0.7)	4.65 (0.6)
MCH:Pg	30.7 (2.5)	30.3 (2.7)	30.8 (2.2)
MCHC: g/dl	35 (1.2)	34.9 (1.5)	35.1 (1.1)
Hct %	40 (5.6)	39.3 (5.4)	40.4 (4.4)
MCV: (Fl)	87 (6.8)	86.4 (6.4)	87.4 (6.7)
WBC x10^3^/µl	5.3 (2.5)	5.4 (2.7)	5.1 (2.5)
Platelet x10^3^/µl	228 (95)	228 (105)	228 (92)

Hb; hemoglobin, RBC: Red blood cell: MCH, Mean Corpuscular; MCHC: Mean Corpuscular Hemoglobin Concentration; MCV: Mean Corpuscular Volume; WBC: White Blood Cell; Hct, Hematocrit; IQR, = Interquartile range. Pg = Picogram, n = Numbers of study participants.

### The magnitude of hematological abnormalities in thyroid hormone dysfunction patients

**Fig 1:**
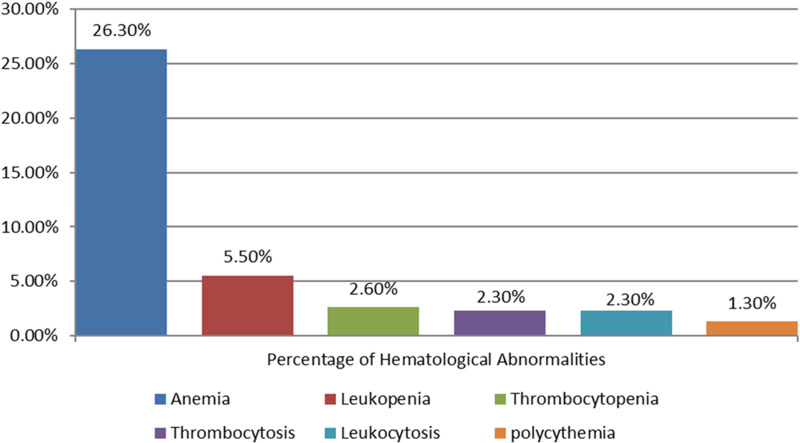
Hematological abnormalities of thyroid dysfunction patients at the University of Gondar Comprehensive Specialized Hospital Northwest, Ethiopia 2022 ( Fig 1). The prevalence of anemia in hypothyroidism was 48 (40.3%), whereas 33 (17.5%) in hyperthyroidism. It was statistically significant with (p-value <0.001). Leukopenia also had statistically significant difference between hypothyroidism 2 (1.7%) and hyperthyroidism 15 (7.9%) (p-value = 0.02). There was no statistically significant association between hypothyroidism and hyperthyroidism in leukocytosis 5 (4.2%) vs 2 (1.1%) (p value = 0.07) (Table 4).

Of the total 308 thyroid dysfunction patients, 81 (26.3%, 95% CI: 21–32%) had anemia. Of the anemic participants, 57 (70.4%) had normocytic normochromic anemia, 23 (28.4%) had microcytic hypochromic anemia, and 1 (1.2%) macrocytic hypochromic anemia. However, polycythemia was seen only in 4 (1.3%, 95% CI: 0.5–3.4) of study participants. Leukopenia and leukocytosis were detected in 17 (5.5%, 95% CI: 3–9%) and 7 (2.3%, 95% CI: 1.1–4.7%) of the respondents, respectively. The overall prevalence of thrombocytopenia was 8 (2.6%) (95% CI:1.3–5.1), and thrombocytosis was observed in 7 (2.3%, 95% CI: 1.1–4.7) of respondents (Fig 1).

**Table 4:** Magnitude of hematological abnormalities and their associations with types of thyroid dysfunction in patients with thyroid hormone dysfunction at the University of Gondar Comprehensive Specialized Hospital, Ethiopia, from March to May 2022 (N = 308).

Hematological abnormalities	Category	Total n (%)	Hypothyroid n (%)	Hyperthyroid n (%)	P-value
Anemia	Yes	81 (26.3)	48 (40.3)	33 (17.5)	0.000^a^
	No	227 (73.7)	71 (59.7)	156 (82.5)	
Types of anemia	Microcytic hypochromic	23(28.4)	16 (33.3)	7 (21.2)	0.32^a^
	Normocytic normochromic	57(70.4)	31 (64.6)	26 (78.8)	0.32^a^
	Macrocytic hypochromic	1 (1.2)	1 (2)	0	0.37^b^
Polycythemia	Yes	4 (1.3)	2 (1.7)	2(1)	0.64^b^
	No	304 (98.7)	117 (98.3)	187(99)	
Leukopenia	Yes	17(5.5)	2(1.7)	15 (7.9)	0.02^b^
	No	291 (94.5)	117 (98.3)	174(92)	
Leukocytosis	Yes	7 (2.3)	5 (4.2)	2 (1.1)	0.07^b^
	No	301 (97.7)	114 (95.8)	187 (98.9)	
Thrombocytopenia	Yes	8 (2.6)	2 (1.7)	6 (3.2)	0.71^b^
	No	300 (96.4)	117 (98.3)	183 (96.8)	
Thrombocytosis	Yes	7 (2.3)	2 (1.7)	5 (2.6)	0.7^b^
	No	301 (97.7)	117 (98.3)	184 (97.4)	

P

^a^ = p-value of Pearson chi2 test P

^b^ = p-value of Fisher’s exact test.

### Factors associated with anemia

In binary logistic regression, hypothyroidism, alcohol consumption, meat consumption, vegetable consumption, and illness (were significantly associated with anemia at a p-value less than 0.25). Accordingly, these variables were included in the multivariable logistic regression model.

In multivariable logistic regression, controlling the co-founding factor, patients with hypothyroidism were 1.95 times (AOR 95% CI:1.0–3.6) more likely to develop anemia than those with hyperthyroidism. The odds of anemia were four times more likely in alcohol consumers than in their counterparts (AOR 95% CI: 1.7–9.2). Those individuals who did not eat meat had four times (AOR95% CI: 1.6–10.4) higher odds of anemia than those who ate meat. Individuals who did not eat vegetables had 2.5 times (AOR 95% CI:1.1–5.5) higher odds of anemia than their counterparts. Respondents with febrile illness were 2.6 times (AOR 95% CI: 1.3–5.4) more likely to develop anemia than those without the febrile disease (Table 5).

**Table 5:** Binary and multivariable logistic regression of anemia determinants in thyroid dysfunction patients at the University of Gondar Comprehensive Specialized Hospital, Ethiopia, from March to May 2022 (n = 308).

Variables	Category	Anemia status n (%)		COR (95%, CI)	P-value	AOR (95% CI)	P value
Anemic	Non-anemic		
Age/years	17–30	9 (11)	27 (12)	1^a^			
	31–45	24 (29.6)	92 (40.5)	0.8 [0.3–2]	0.296	1.02 [0.9–1.0]	0.07
	> 45	48 (59.3)	108 (47.6)	1.3 [0.6–3]	0.002	1.1[0.9–1.1]	0.06
Sex	Male	8 (10)	25 (11)	1^a^			
	Female	73 (90)	202 (89)	1.13 [0.5 - 2.6]	0.005	0.5[0.2–1.2]	0.13
Marital statues	Married	36 (44.4)	122 (53.7)	1^a^			
	Unmarried	17 (21)	42 (18.5)	1.3 [0.7–2.7]	0.359	1.7 [0.7–4.0]	0.23
	Divorced	22 (27.2)	42 (18.5)	1.8 [0.9–3.4]	0.037	2.4 [1.0–5.6]	0.064
	Widowed	6 (7.4)	21 (9.2)	1 [0.4–2.6]	0.68	1.06[0.3–3.3]	0.9
Educational statues	Unable to read and write	47 (58)	118 (52)	1.4 [0.3–6]	0.504	1.3 [0.5–3.1]	0.58
	Primary school	18 (22.2)	56 (24.7)	1.1 [0.2–6]	0.03	0.5 [0.3–2.7]	0.83
	Secondary school	14 (17.3)	46 (20.3)	1 [0.25–7]	0.443	1.4 [0.2–11.6]	0.71
	Certificate and above	2 (2.47)	7 (3)	1^a^			
Residence	Rural	53 (65.4)	148 (64)	1 [0.6–1.8]	0.003	1.01[0.4–2.0]	0.979
	Town	28 (34.6)	82 (36.1)	1^a^			
Thyroid dysfunction	Hypothyroidism	48 (40.3)	71 (59.7)	3.19 [1.9–5.4]	0.002	1.95[1.1–3.6]	0.030
	Hyperthyroidism	33 (17.5)	156 (82.5)	1^a^		1^a^	
Alcohol	Yes	25 (56.82)	19 (43.2)	4.9 [2.5–9.5]	0.001	4.0 [1.7–9.2]	0.001
	No	56 (21.2)	208 (78.8)	1^a^		1^a^	
Meat consumption	Yes	7 (6.8)	96 (93.2)	1^a^		1^a^	
	No	72 (35.5)	131 (64.5)	7.5 [3.3–17]	0.001	4.0 [1.6–10.4]	0.004
Vegetable	Yes	12 (9.5)	114 (90.5)	1^a^		1^a^	
	No	68 (37.6)	113 (62.4)	5.7 [2.9–11.1]	0.001	2.5 [1.1–5.5]	0.024
Febrile illness	Yes	32 (56.1)	25 (43.9)	5.3 [2.9–9.7]	0.001	2.6 [1.3–5.4]	0.009
	No	49 (19.5)	202 (80.5)	1^a^		1^a^	

1

^a^ = Reference category, AOR = adjusted odds ratio, OR = crude odds ratio, CI = confidence interval.

## Discussion

Previous studies have demonstrated that thyroid hormone dysfunction has a significant impact on blood cell parameters [11,31,32]. However, hematological abnormalities, as well as the associated factors in patients with thyroid hormone dysfunction, have not been thoroughly explored. Therefore, this study aims to identify hematological abnormalities and their associated factors in patients with thyroid hormone dysfunction.

The current study found that 26.3% (95% CI: 21–32%) of the participants had anemia. Based on the World Health Organization classification, this indicates that anemia represents a moderate public health issue among patients with thyroid dysfunction [25]. It was higher than the prevalence of anemia in the general population [33]. Moreover, the prevalence of this finding was in line with the studies conducted in Kenya (28.4%) [34] and Iraq (31.3%) [35].

However, it was higher than studies conducted in New York (5.9%) [36], Switzerland (5.9–6.54%) [37,38], Italy (7.5–12%) [39,40], Spanish 18.6% [41], UK (4.2%) [42]. On the other hand, It was lower than studies done in Saudi Arabia (36.37–60.27%) [18,43,44], Turkish (41%) [45], Tunisia (53.5%) [46] and India (57–79.5%) [47,48]. The possible explanation for the difference could be attributed to variations in the socio-demographic characteristics, physiological, local prevalence of parasitic infections, dietary habits, and a factor that determines nutrition and health-being [49–52].

The incidence of anemia can be scientifically explained by the role of thyroid hormones, which interact with nuclear receptors known as thyroid receptor α (TRα) and thyroid receptor β (TRβ). These receptors exhibit significant polymorphism, affecting their function and influence on various physiological processes. Furthermore, thyroid hormones promote the binding of nuclear receptor co-activator 4 to chromatin regions near RNA polymerase II (Pol II), a process closely associated with transcription during terminal differentiation. This complex mechanism highlights the connection between thyroid function and hematopoiesis, emphasizing how changes in thyroid hormone levels may contribute to the development of anemia [53]. Specifically, genetic polymorphisms in the TRβ locus are associated with abnormal hematological traits [54]. Thyroid hormone is essential for the terminal differentiation of human erythroid progenitors, a process primarily mediated by thyroid receptor β (TRβ). In vitro studies have shown that the specific removal of TRβ from the culture medium completely halts terminal erythroid differentiation and enucleation. This highlights the critical role of TRβ in red blood cell (RBC) production; any defects in TRβ can disrupt erythropoiesis, potentially leading to anemia [53]. Another potential mechanism involves the crucial role of cytokines in regulating immune responses. Inflammatory cytokines can directly or indirectly suppress erythropoiesis through various pathways: they may reduce erythropoietin production, decrease the sensitivity of erythroid progenitors to erythropoietin, and exert direct inhibitory effects on erythroid precursors. Moreover, these cytokines can shorten the lifespan of erythrocytes, thereby exacerbating anemia [55]. In this study, the most prevalent types of anemia were normocytic normochromic anemia (70.4%), followed by microcytic hypochromic anemia (28.4%) and macrocytic normochromic anemia (1.2%). These findings are consistent with studies conducted in Poland, Turkey, and India [45,56,57]. In contrast, studies conducted Iraq reported that microcytic anemia was the most common type of anemia [58]. Most studies indicate that normocytic and normochromic anemia are the most prevalent types of anemia. This could be because thyroid disease often leads to bone marrow suppression, which in turn may decrease erythropoietin production. Normocytic normochromic anemia seems to arise from reduced RBC production caused by underlying conditions like thyroid dysfunction [59,60]. Additionally, microcytic anemia is linked to iron malabsorption and iron loss due to menorrhagia. On the other hand, macrocytic anemia is associated with thyroid dysfunction, which can impair the absorption of vitamin B12 and folate, leading to reduced oxygen metabolism.

According to the present study, leucopenia was the second most common hematological abnormality observed in 5.5% (95% CI: 3–9%) of patients with thyroid disease. This finding was in line with the study done in Poland that reported 8.5% leukopenia in patients with thyroid hormone disorder [61]. However, it is lower than the study findings in Kenya, 12.2% [62]. These variations can be due to socio-demographic and behavioral characteristics, the prevalence of infections, inflammatory disease, and dietary habits. The specific mechanisms of leukopenia, such as marrow suppression and anti-neutrophil antibodies, were also unclear and controversial [63,64]. However, some studies suggest that the possible mechanism might be thyroid-stimulating antibodies found in Grave’s disease inhibit the production of white blood cells [65].

On the other hand, patients with Hashimoto’s thyroiditis have a high degree of circulating immune complexes, function, and proliferation [66]. Thyroid hormone deficiency promotes the initiation of programmed cell death. The excessive production of reactive oxygen species and compromised integrity of the outer mitochondrial membrane trigger apoptosis. Reactive oxygen species lead to lipid peroxidation in cell membranes, low-density Lipoproteins, and the mitochondrial membrane, causing the release of cytochrome C and activation of caspase-3, ultimately resulting in apoptosis [67,68].

In contrast, leukocytosis was seen in around 2.3% (95% CI: 1.1–4.7%) of patients. This was in agreement with a study in Iraq of 4.3% [58]. The result shows that thyroid dysfunction also secrets colony-stimulating factors that induce leukocytosis [69]. Due to the incomplete understanding of thyroid hormone mechanisms, many reported hematological changes remain inadequately explained. However, recent advancements have shed light on thyroid hormone action at the cellular level. In hormone-responsive tissues, T3 initiates hormonal action through nuclear receptors identified as non-histone proteins. These receptors are closely related to the products of cellular proto-oncogenes and belong to a receptor family that includes those for steroid hormones, vitamin D, and retinoic acid. Once bound, the T3-receptor complex is believed to attach to specific DNA regions, thereby modifying gene expression [70,71]

Thrombocytopenia was observed in 2.6% (95% CI:1.3–5.1) of the study participants. The current finding is in agreement with previous studies conducted in Kenya (4.7%) [62], Japan (4.6%) [72], Iraq (4%) [58], and Poland 5% [61]. However, it is lower than studies done in California (43%) [73,74]. Another theory proposes a cross-reaction between the antibody’s anti-thyroid receptors and the platelet epitopes, which could explain the association of both conditions. This idea was strengthened by the structural similarity of the platelet membrane glycoprotein and the truncated actin-binding protein, which is the protein that anti-thyroid antibodies bind [75]. This suggests that the same anti-thyroid antibodies react with platelet receptors leading to platelet destruction [76].

This study detected thrombocytosis in 2.3% (95% CI: 1.1–4.7) of study participants. This finding agreed with a study done in Iraq (2%) [58].These variations may be due to socio-demographic and behavioral characteristics, the prevalence of infections, disease conditions, and dietary habits. The possible mechanism of thrombocytosis may be thyroid dysfunction, which leads to increased platelet production. The previous findings explained that platelet lifespan is slightly reduced in hyperthyroid patients. Therefore increased thrombopoiesis result from the shortened platelet lifespan to maintain a normal platelet count [26].

The least common hematological abnormalities were polycythemia observed in 1.3% (95% CI: 0.5–3.4). Erythrocytosis may appear in patients with hyperthyroidism, and one possible mechanism is thyroid hormone-induced augmentation of hypoxia-inducible factor1, resulting in increased erythropoietin levels [77]. The erythro-kinetic study also indicated heightened erythropoietic activity in the bone marrow. It is hypothesized that thyroid hormones stimulate erythropoiesis, which can sometimes result in erythrocytosis, provided there is no deficiency in hematopoietic nutrients. This stimulation of erythropoiesis by thyroid hormones is thought to be mediated through erythropoietin. In many hyperthyroid patients, an increased erythrocyte mass is observed, likely due to enhanced production during the early stages of erythrocyte development [78,79].

Hypothyroidism patients were 1.95 times (AOR 1.95, 95% CI: 1.0, 3.6) more likely to develop anemia than hyperthyroidism patients. Similarly, studies showed that anemia is more common in hypothyroidism patients [43,58,80]. This may be because hypothyroidism directly affects the process of hematopoiesis by influencing erythroid precursor Proliferative capacity. And also by suppressing the activity of bone marrow and the tissue that makes RBC [43,80]. Moreover, Hypothyroidism may have indirect effects on erythropoiesis by reducing gene expression and secretion of erythropoietin from the kidney [78,81].

This finding also revealed that alcohol consumption was the risk for the prevalence of anemia among thyroid dysfunction patients. The odds of anemia were four times (AOR 95% CI: 1.7, 9.2) higher among thyroid patients who had taken alcohol than who hadn’t taken alcohol. One possible reason for this effect might be that alcohol consumption impacts iron absorption. Alcohol interferes with an enzyme crucial for a key step in hemoglobin synthesis and can also be directly toxic to the bone marrow. In alcoholics, there is often reduced erythrocyte production in the bone marrow and a shorter RBC survival time. The high prevalence of macrocytosis and macrocytic anemia among alcoholics may be attributed to hematopoietic system impairment and an increased rate of erythrocyte destruction due to severe exposure to acetaldehyde [82–85].

Individuals who did not consume meat had a fourfold (AOR 95%CI: 1.6, 10.4) increased risk of developing anemia compared to those who did consume meat. This issue might stem from insufficient iron intake, particularly from heme iron found primarily in meat, which can lead to iron deficiency anemia. Higher concentrations of hemoglobin are associated with the consumption of red meat, a crucial source of heme iron. Reports indicate that meat plays a significant role in enhancing iron absorption through the diet [86,87].

Respondents who did not consume vegetables had 2.5 times higher odds of anemia (AOR 95% CI 1.1–5.5) compared to those who did, after adjusting for other factors. This is likely because vegetables are a valuable source of non-heme iron and also provide Vitamin C, which enhances the absorption of non-heme iron from legumes and other plant-based foods. A deficiency in Vitamin C can lead to hemolysis, causing damage and destruction of erythrocytes [88,89].

Respondents with febrile illness were 2.6 times more likely to develop anemia (AOR 95% CI 1.3–5.4) compared to those without fever. This may be due to immune activation from infection, which triggers the release of cytokines such as tumor necrosis factor, interleukin-1, gamma-interferon, and beta-interferon. These cytokines inhibit colony-forming units-erythroid and stimulate the production of hepcidin from the liver, which in turn blocks ferroportin. Ferroportin regulates iron export from the gut and iron-storing cells (macrophages). Consequently, circulating iron levels decrease, reducing duodenal iron absorption and macrophage iron recycling. This leads to iron deficiency in the serum, impairing erythroid cell proliferation and hemoglobin synthesis [90–92]. When assessing the results of this study, it is important to take into account both its strengths and limitations. This study is One of the earliest laboratory-based investigations to ascertain the extent and contributing variables of hematological abnormalities in individuals with thyroid dysfunction. However, It was not without limitation. First, the capacity to identify intestinal parasites may be diminished because stool testing was limited to the saline wet mount approach. Second, recall bias may exist because responses to the questions for some variables were based on recall knowledge. Third, some of the questions, including alcohol and tobacco use, can be understated due to social desirability bias. Fourth, analysis of RBC morphology and research into the precise causes of anemia, including levels of iron, vitamin B12, and folate were not performed, Lastly, this study was unable to determine any causal links due to its cross-sectional design.

## Conclusion and recommendation

### Conclusion

According to the current study, anemia was the predominant hematological abnormality. Therefore, thyroid hormones have a significant influence on erythropoiesis. It was a mild public health problem in patients with thyroid disease. Its prevalence was significantly higher among hypothyroidism patients compared to hyperthyroidism patients. The most common types of anemia were normocytic normochromic anemia, whereas macrocytic anemia was found to be on the list. Leukopenia was the second predominant hematological abnormality, followed by thrombocytopenia. Alcohol consumption, vegetable consumption, meat consumption, and febrile illness were also associated with anemia in the current study.

### Recommendation

According to the finding, we suggested that patients with thyroid dysfunction should have regular hematological test monitoring for possible hematological changes. All patients diagnosed with hematological abnormalities should be evaluated for thyroid disorders before beginning treatment. Clinicians should routinely check the hematological abnormalities of patients with thyroid dysfunction. Moreover, researchers and reviewers should focus on carefully investigating specific hematological abnormalities in thyroid dysfunction patients. That would result in significant findings and contribute to understanding how thyroid hormones affect hematological parameters. The government should also emphasize and encourage funding medical facilities to enhance the treatment provided to patients with thyroid dysfunction.

## Supporting information

S1 Data Data set. (XLSX)

S1 File Supporting information. (DOCX)

## Abbreviations and acronyms:

CBC: complete blood count
Hb: hemoglobin
Hct: hematocrit
MCH: mean corpuscular hemoglobin
MCHC: mean corpuscular hemoglobin concentration
MCV: mean corpuscular volume
MPV: mean platelet volume
PDW: platelet distribution width
RBCs: red blood cells
RDW: red cell distribution width
SOP: standard operational procedure
T3: triiodothyronine
T4: thyroxin
TSH: thyroid stimulating hormone
TSHR: thyroid-stimulating hormone receptor
TRα: thyroid hormone nuclear receptors α
TRβ: thyroid hormone nuclear receptors β
WBC: white blood cells
