# Supplementary material for: Hematological abnormalities and associated factors among patients with thyroid hormone dysfunction at the University of Gondar Comprehensive Specialized Hospital, Northwest Ethiopia
Source: PLoS One. 2025 May 2;20(5):e0322748. doi: 10.1371/journal.pone.0322748 (PMC12047792; doi:10.1371/journal.pone.0322748)
Supplement: S1 File — (DOCX) [file pone.0322748.s002.docx]

Information sheet English version

Study title: Cross-sectional study on hematological abnormalities in patients with thyroid hormone dysfunction at University of Gondar Specialized referral hospital North west Ethiopia.

You have been invited to take part in this research study. Before you decide whether to take part it is important for you to understand why the research is being done and what it will involve. Thyroid disease is a medical condition that affects the function of the [thyroid gland.](https://en.wikipedia.org/wiki/Thyroid) The thyroid gland is located at the front of the neck and produces [thyroid hormones.](https://en.wikipedia.org/wiki/Thyroid_hormones) That travel through the blood to help regulate many other organs. These hormones normally act in the body to regulate energy use, infant development, and childhood development. There will be little pain during sample collection. The purpose of the study, I am evaluating the hematological profile in patients with thyroid dysfunction.

**Consent form**

I have read the information above, or it has been read to me. I have been given the opportunity to ask questions and my questions have been answered to my satisfaction. I voluntarily consent that I would participate in this study. To collect my blood and be a participant in this study and understand that I have the right to withdraw from the study at any time.

_______________________________________ ____ /____ /____ (dd/mm/yy)

If illiterate;

Print name of independent literate witness, date and signature of witness (if possible, this person should be selected by the participant and should have no connection to the research team)

____________ ____/____/_____ (dd/mm/yy) Phone number: ____________________

Print name of researcher, date and signature of researcher ______ ______/____/_____ (dd/mm/yy

Part 1: socio demographic characteristics related questionnaires.

| SN | QUESTIONS | RESPONSE |
| --- | --- | --- |
| 1. | Sex | 1) Female 2) Male |
| 2. | Age (years) | ________________ |
| 3. | Marital status | 1)Married 2) Single  3)Divorced 4) Widowed |
| 4. | Occupation | 1) Civil servants 2) House wife  3) Private organization 4) Farmer  5) Daily laborer 6) Merchant  7. Others |
| 5. | Residence | 1.Rural 2. Urban |
| 6. | Educational status | 1.Unable to read and write 2. Primary school  3.High school 4. Certificate and above |

**Part 2: Behavioral and feeding related questionnaires**

| 1. | Smoking status | 1.smoker----------- 2. Nonsmoker--------- |
| --- | --- | --- |
| 2. | Alcohol consumption | 1 Alcoholics ------- 2 Non alcoholics---------- |
| 3. | Do you use Iodine based salt? | 1.Yes: ----------- 2. No--------------- |
| 4. | Do you use meat in your diet? | 1.Yes-------------- 2. No--------------------- |
| 5. | Do you use Vegetable’s in your diet? | 1yes ………. 2. No…………. |

**Part 3: Clinical characteristics related questionnaires**

| 1. | Do you have febrile illnesses | 1.Yes -------2. No ------ |
| --- | --- | --- |
| 2 | Type of Thyroid dysfunction | 1.Hypothyroidism,  2. Hyperthyroidism |
|  | Should be filled by laboratory technologist |  |
| 3. | Stool examination | 1. No ova of parasites seen……  2. ova of parasite seen……  Types of parasites seen ………. |
| 4. | Blood film examination | 1.No-hemoparasite seen …  2. Hemoparasite seen ……  Types of hemoparasites seen ………. |

## Part 4: Hematological profiles of thyroid dysfunction patients.

| **Parameters** | **Total (n=308)** | **Hypothyroidisms (n=119)** | **Hyperthyroidisms (n=189)** |
| --- | --- | --- | --- |
|  | **Median (IQR)** | **Median (IQR)** | **Median (IQR)** |
| Hb: g/dl | 14 (2.2) | 13.6 (2.3) | 14.4 (1.5) |
| RBC:10^6^/µl | 4.63 (0.6) | 4.6 (0.7) | 4.65 (0.6) |
| MCH:Pg | 30.7 (2.5) | 30.3 (2.7) | 30.8 (2.2) |
| MCHC: g/dl | 35 (1.2) | 34.9 (1.5) | 35.1 (1.1) |
| Hct % | 40 (5.6) | 39.3 (5.4) | 40.4 (4.4) |
| MCV: (Fl) | 87 (6.8) | 86.4 (6.4) | 87.4 (6.7) |
| WBC x10^3^/µl | 5.3 (2.5) | 5.4 (2.7) | 5.1 (2.5) |
| Platelet x10^3^/µl | 228 (95) | 228 (105) | 228 (92) |

**Part 5:** The magnitude of hematological abnormalities in thyroid hormone dysfunction patients.

| **Hematological abnormalities** | **Category** | **Total n (%)** | **Hypothyroid n (%)** | **Hyperthyroid n (%)** | **P-value** |
| --- | --- | --- | --- | --- | --- |
| \|  \|  \|  \|  \|  \| \| --- \| --- \| --- \| --- \| --- \|   Anemia | Yes | 81 (26.3) | 48 (40.3) | 33 (17.5) | 0.000 ^a^ |
|  | No | 227 (73.7) | 71 (59.7) | 156 (82.5) |  |
| Types of anemia | Microcytic hypochromic | 23(28.4) | 16 (33.3) | 7 (21.2) | 0.32 ^a^ |
|  | Normocytic normochromic | 57(70.4) | 31 (64.6) | 26 (78.8) | 0.32 ^a^ |
|  | Macrocytic hypochromic | 1 (1.2) | 1 (2) | 0 | 0.37 ^b^ |
| Polycythemia | Yes | 4 (1.3) | 2 (1.7) | 2(1) | 0.64 ^b^ |
|  | No | 304 (98.7) | 117 (98.3) | 187(99) |  |
| Leukopenia | Yes | 17(5.5) | 2(1.7) | 15 (7.9) | 0.02 ^b^ |
|  | No | 291 (94.5) | 117 (98.3) | 174(92) |  |
| Leukocytosis | Yes | 7(2.3) | 5(4.2) | 2(1.1) | 0.07 ^b^ |
|  | No | 301 (97.7) | 114 (95.8) | 187 (98.9) |  |
| Thrombocytopenia | Yes | 8 (2.6) | 2(1.7) | 6 (3.2) | 0.71 ^b^ |
|  | No | 300(96.4) | 117(98.3) | 183 (96.8) |  |
| Thrombocytosis | Yes | 7(2.3) | 2(1.7) | 5(2.6) | 0.7 ^b^ |
|  | No | 301(97.7) | 117 (98.3) | 184(97.4) |  |

## Part 6: Factors associated with anemia.

| Variables | Category | Anemia status n (%) | | COR (95%, CI) | P-value | AOR (95% CI) | P value |
| --- | --- | --- | --- | --- | --- | --- | --- |
|  |  | Anemic | Non-anemic |  |  |  |  |
| Age/years | 17-30 | 9 (11) | 27 (12) | 1^a^ |  |  |  |
|  | 31-45 | 24 (29.6) | 92 (40.5) | 0.8 [0.3-2] | 0.296 | 1.02 [0.9 - 1.0] | 0.07 |
|  | > 45 | 48 (59.3) | 108 (47.6) | 1.3 [0.6-3] | 0.002 | 1.1[0.9- 1.1] | 0.06 |
| Sex | Male | 8 (10) | 25 (11) | 1^a^ |  |  |  |
|  | Female | 73 (90) | 202 (89) | 1.13 [ 0.5 - 2.6] | 0.005 | 0.5[ 0.2- 1.2] | 0.13 |
| Marital statues | Married | 36 (44.4) | 122 (53.7) | 1^a^ |  |  |  |
|  | Unmarried | 17 (21) | 42 (18.5) | 1.3 [0.7-2.7] | 0.359 | 1.7 [o.7- 4.0] | 0.23 |
|  | Divorced | 22 (27.2) | 42 (18.5) | 1.8 [0.9-3.4] | 0.037 | 2.4 [1.0-5.6] | 0.064 |
|  | Widowed | 6 (7.4) | 21 (9.2) | 1 [0.4-2.6] | 0.68 | 1.06[0.3-3.3] | 0.9 |
| Educational statues | Unable to read and write | 47 (58) | 118 (52) | 1.4 [0.3-6] | 0.504 | 1.3 [0.5- 3.1] | 0.58 |
|  | Primary school | 18 (22.2) | 56 (24.7) | 1.1 [0.2-6] | 0.03 | 0.5 [0.3-2.7] | 0.83 |
|  | Secondary school | 14 (17.3) | 46 (20.3) | 1 [0.25-7] | 0.443 | 1.4 [0.2-11.6] | 0.71 |
|  | Certificate and above | 2 (2.47) | 7(3) | 1^a^ |  |  |  |
| Residence | Rural | 53(65.4) | 148(64) | 1[0.6-1.8] | 0.003 | 1.01[0.4 – 2.0] | 0.979 |
|  | Town | 28(34.6) | 82(36.1) | 1^a^ |  |  |  |
| Thyroid  Dysfunction | Hypothyroidism | 48 (40.3) | 71(59.7) | 3.19 [1.9-5.4] | 0.002 | 1.95[1.1-3.6] | 0.030 |
|  | Hyperthyroidism | 33 (17.5) | 156 (82.5) | 1^a^ |  | 1^a^ |  |
| Alcohol | Yes | 25 (56.82) | 19(43.2) | 4.9 [2.5- 9.5] | 0.001 | 4.0 [1.7 - 9.2] | 0.001 |
|  | No | 56(21.2) | 208 (78.8) | 1^a^ |  | 1^a^ |  |
| Meat consumption | Yes | 7 (6.8) | 96(93.2) | 1^a^ |  | 1^a^ |  |
|  | No | 72 (35.5) | 131 (64.5) | 7.5 [3.3-17] | 0.001 | 4.0 [1.6 -10.4] | 0.004 |
| Vegetable | Yes | 12 (9.5) | 114(90.5) | 1^a^ |  | 1^a^ |  |
|  | No | 68(37.6) | 113(62.4) | 5.7 [2.9-11.1] | 0.001 | 2.5 [1.1 -5.5] | 0.024 |
| Febrile illness | Yes | 32 (56.1) | 25(43.9) | 5.3 [2.9-9.7] | 0.001 | 2.6 [1.3-5.4] | 0.009 |
|  | No | 49 (19.5) | 202 (80.5) | 1^a^ |  | 1^a^ |  |

Amharic version

የመረጃ ቅጽ አማርኛ

የጥናቱ ዓላማ በታይሮይዴ እጢ (እንቅርት) ተጠርጣሪ ህመምተኞች የደምሴሎች መጠን ላይ የሚታዩ ለዉጦችን በጎንደር ሆስፒታል ውስጥ ምርምር ማድረግ: በጥናቱ ላይ ከመሳተፍዎ በፊት የጥናቱን አስፈላጊነት መረዳት ያሰፈልገዎታል የታይሮይድ መጠን መጨመር እና ትክክኛዉን ተግባሩን ማከናወን በመቸገር ምክንያት የአንገት ወይም የጉሮሮ ማበጥ እንዲከሰት የሚያደርግ የጤና እክልነዉ፡፡ ከዚህም በተጨማሪ በሽታዉ የታይሮይድ ሆርሞኖች በብዛት ወይም ከሚፈለገዉ በታች መመረት በሚፈጥረዉ ችግር ምክንያት የሚከሰት ነዉ፡፡ የዚህ ጥናት ዉጤት ስለበሽታው የተሻለ እውቀት እንዲያገኙ እንዲሁም በሽታው ከደምሴሎች ጋር ያለዉን ቁርኝት ማወቅ በጊዜ ለመለየትና ለመከላከል ለሚደረገዉ ጥረት ጉልህ ድርሻ ይኖረዋል፡፡

ለአስተያየትዎ

ዋና ተመራማሪ: በፍቃድ ማንደፍሮ

ስልክ: 0932497185

ኢሜል: [**befikadm@gmail.com**](mailto:befikadm@gmail.com)

**10.1 የስምምነት መጠየቂያ ቅጽ**

ተሳታፊ የሚፈርሙት የስምምነት ቅጽ የጥናቱን አላማና ሂደት በዝርዝር ከተረዱ በኃላ የሚከተለውን ቅጽ በጥንቃቄ ይፈርማሉ

የጥናቱ ተሳታፊ እንድሆን በሙሉ ፈቃዴ ወስጃለሁ ከዚህጋር የተያያዘውን የመግለጫ ቅጽ በትክክል አንብቤ ተረድቻለሁ፡፡ በእኔላይም ስለሚደረግ ማንኛውም ጥናት ተገንዝቤ አለሁ፡፡በተጨማሪም አስፈላጊውን ገለጻና ማብራሪያ ከላይ በተጠቀሱት ሰው ተደርጎልኛል፡፡ ትንሽ የደም ናሙና እንደሚወስድ በሚገባ ተረድቻለሁ፡፡ አጥኚዎቹ የደም ምርመራ ውጤቴን ወስደው ይጠቀማሉ በተጨማሪም ጥያቄ የመጠየቅ መብት የመወያየት እድል ከላይ ከተጠቀሱት አጥኚዎች ወይም ከነሱ ተወካይ ጋር ተሰጥቶኝ በጥናቱ ላይ በቂ ምክርና ውይይት አዴርጌያለሁ፡፡ በተመራማሪዎቹ የጥናቱን ውጤት ይፋ እንዱያደርጉ እፈቅዳለሁ፡፡ ነገርግን ስም መጠቀስ የለበትም: :ተመራማሪዎቹ በጤናዬ ላይ ያለውን ችግር እንዲነግሩኝ ፈቅጀላቸዋለሁ:: በማንኛውም ጊዜ ከጥናቱ እራሴን ማግለል እንደምችል አውቂያለሁ፡፡ ከእኔ የሚሰበሰበው ማንኛውም መረጃ በጥንቃቄና ሚስጥራዊነቱ በተጠበቀ ቦታ እንደሚቀመጥ አውቄያለሁ፡፡

ፊርማ_____________________ ቀን_______________________ ይህ በጥናቱ የሚሳተፈው ሰው መፈረም ስለማይችል ከላይ የተዘረዘሩት መረጃዎች ለተሳታፊው የተሰጡና ተሳታፊውም ለመሳተፍ መስማማቱን ገለልተኛ ታዛቢ በመሆን አረጋግጣለሁ፡፡ ________________________ __________________________ የገለሌተኛ ታዛቢ .ፊርማ…………………….ቀን………………..ስለጥናቱ ዝርዝር መረጃ ስለመስጠቴ አረጋግጣለሁ ……………………………………………………… የመተማመኛ ቅጹን ከስምምነት ቅጽ ጋር አያይዤ ሰጥቻለሁ ፊርማ……………………………

**10.2 ማህበራዊ ነክ እና ተያያዥ ጥያቄዎች**

ክፍል -1

| ተቁ | መጠይቆች | መልስ |
| --- | --- | --- |
| ማህበራዊ ሁኔታ | |  |
| 1. | ፆታ | 1.ወንድ --- 2. ሴት--- |
| 2. | እዴሜ |  |
| 3. | የጋብቻ ሁኔታ | 1. ያገባ/ባች -----2. ያላገባ/ባች----  3. የተፋታ/ች------4. በሞትየተለየ--- 5 ……ሌላ------- |
| 4. | የስራ ሁኔታ | 1.አርሶ አደር------2. የመንግስት ሰራተኛ  3.የቤት እመቤት------4. የግል  5. የቀንሰራትኛ----6. ነጋዴ  7.ሌላ ……………… |
| 5. | መኖሪ ያቦታ | 1.ገጠር--------2. ከተማ--------- |
| 6. | የትምህርት ደረጃ | 1 ያልተማረ/ች ----2. አንደኛ ደረጃ  3.ሁለተኛ ደረጃ-------3. ሰርትፍኬት እና ከዚያ በላይ |
| 7. | ሲጋራ ያጨሳሉ | አዎ------2. አላጨስም |
| 8. | በቤት ዉስጥ አዮዲን ጨዉ ይጠቀማሉ | 1.አዎ ------2. አልጠቀምም |
| 9. | አልኮሆል ትጠጣለህ/ሽ | 1 አዎ 2 አልጠጣም |
| 10. | በአመጋገብዎ ውስጥ ስጋን ይጠቀማሉ? | አዎ 2 አልጠቀምም |
| 11 | 12. በአመጋገብዎ ውስጥ አትክልት ይጠቀማሉ? | 1. አዎ 2. አልጠቀምም |

**10.3 ህክምና ነክ የታይሮይ ህመም እና ከደም ሴል ጋር ተያያዥ ጥያቄዎች**

**ክፍል -2**

| ተ.ቁ | **ጥያቄ** | መልስ |
| --- | --- | --- |
| 1. | የትኩሳት አለብዎት | 1.አዎ----------2. የለኝም |
| 2. | የታይሮይድ ህመሙ አይነት | 1.ሐይ ፖታይሮይዲዝም  2. ሐይፐርታይሮይዲዝም |
|  | **በቤተ ሙከራ ቴክኖሎጂ ባለሙያ የሚሞላ** |  |
| 3 | ጥገኛ ኢንፌክሽን (parasitic infections) | 1……………………………….2……………… |
| 4 | የደም ፊልም (blood film) | 1……………………………2…………………… |
